# Monitoring Internal Load in Women’s Basketball via Subjective and Device-Based Methods: A Systematic Review

**DOI:** 10.3390/s23094447

**Published:** 2023-05-02

**Authors:** Javier Espasa-Labrador, Azahara Fort-Vanmeerhaeghe, Alicia M. Montalvo, Marta Carrasco-Marginet, Alfredo Irurtia, Julio Calleja-González

**Affiliations:** 1 INEFC-Barcelona Research Group on Sport Sciences (GRCE), National Institute of Physical Education of Catalonia (INEFC), University of Barcelona (UB), 08038 Barcelona, Spain; 2 FPCEE and FCS Blanquerna, SAFE Research Group, Ramon Llull University, 08022 Barcelona, Spain; 3 Segle XXI Female Basketball Team, Catalan Federation of Basketball, 08915 Esplugues de Llobregat, Spain; 4 College of Health Solutions, Arizona State University, Phoenix, AZ 85004, USA; 5 Department of Physical Education and Sport, Faculty of Education and Sport, University of the Basque Country, (UPV/EHU), 01007 Vitoria, Spain; 6 Faculty of Kinesiology, University of Zagreb, 10110 Zagreb, Croatia

**Keywords:** physiological response, monitoring, female basketball

## Abstract

The monitoring of internal load in basketball can be used to understand the effects and potential physiological adaptations caused by external load. The main aim of this systematic review was to identify the methods and variables used to quantify internal load in female basketball. The studies included different populations and events: youth athletes, elite, and amateur players. Subjective methods included using the rating of perceived exertion (RPE) method, and sensor-based methods included monitoring the cardiac response to exercise, using heart rate (HR) as the primary metric. The results showed that the HRAvg exhibited a wider range of values during training than during competition, and different metrics were used to evaluate internal load, such as HRMax, HRmin, %HRMax, total time and % of time spent in different HR zones (2–8 zones), Banister’s TRIMP, and summated HR zones. RPE and HR metrics were the most commonly used methods. However, the use of multiple metrics with little standardization resulted in significant heterogeneity among studies, limiting meaningful comparisons. The review provides a reference for current research on female basketball. Future research could address this limitation by adopting more consistent measurement protocols standardizing the use of metrics.

## 1. Introduction

With the ever-increasing demand for optimal performance in basketball, researchers and clinicians are constantly striving to discover new ways to gain a competitive edge, leading to significant advancements in the field of basketball science [1]. Recently, there has been an increase in interest in managing and monitoring internal and external load to reduce injury risk and enhance performance [2]. There are two types of loads: (1) external load, or the amount of work done in a time period or bout of activity, and (2) internal load, or the psycho-physiological response to external load. Because external load is task-dependent, it is position-dependent in sport. Conversely, internal load depends on the psycho-physiologic and intrinsic factors in the athlete, such as motivation, stress, fatigue, cognitive capacity, age, gender, sport experience, and physical condition [3]. Due to the intermittent nature of team sports, it is imperative to understand internal load and quantify its relationship with a dose-response of activity for precise training planning [4]. The most common methods for quantifying internal load in team sports are biochemical parameters (e.g., blood lactate concentration [BLC]), oxygen consumption (VO2), heart rate (HR) activity, and the rating of perceived exertion (RPE) [5]. Among these, HR activity and RPE can be assessed through various metrics, such as Banister’s training impulse (TRIMP_B_) and Edward’s summated heart rate zones score (SHRZ) for HR activity, and Foster’s proposal, multiplying session RPE by session time (sRPE) for RPE. These metrics express their data in arbitrary units (AU), and provide a quantitative measure of the internal load experienced by the athlete, allowing for a more precise assessment of training adaptations and injury risk.

The existing literature has described methods for quantifying internal load in a range of team sports [6], including girl’s and women’s basketball. Research on internal load in sports aims to improve understanding of the impact of internal load and individual physiological responses to different stimuli. This understanding can maximize positive training adaptations, minimize the negative effects of training, and enhance performance. However, the majority of research on internal load has focused on male basketball players [7], and more research is needed on female basketball players. In recent years, exercise physiology research has emphasized the physiological characteristics and differences between women and men [8]. However, there is a dearth of scientific evidence on the internal response of female athletes to various sports, including basketball [6,9]. It is crucial to identify the most effective methods for evaluating internal load in female basketball players of different competitive levels and age groups to improve training planning and load management. Despite the existence of systematic reviews on internal load monitoring methods in female basketball players [10,11], there is no previous review on the techniques used to measure or report internal load in this population.

The primary objective of this systematic review is to identify the common methods and metrics used to quantify internal load in female basketball players during training and competition, by level of competition and age. The secondary objectives are to identify potential normative values and critique the heterogeneity of the methods used.

## 2. Materials and Methods

### 2.1. Search Strategy

This is a systematic review focused on monitoring physiological responses during training and competition in female basketball players at varying levels of competition. The review was conducted according to the Preferred Reporting Items for Systematic Reviews and Meta-Analysis protocols (PRISMA-P^®^) statement [12].

A structured search was carried out in the EBSCO, PubMed, Scopus, and Web of Science. The search encompassed all articles published until 31 January 2023. The following Boolean search equation was used to find the relevant articles: (“female” OR “woman”) AND “basketball” AND (“monitoring” OR “training” OR “internal” OR “physiological”) AND “load”. The search strategy was modified for the PubMed database, namely through the use of medical subject headings (MeSH) and free-text words for key concepts related to the monitoring of load in the training or competition of the athletes under investigation. The specific search strategy used in PubMed was: ((“female” [MeSH Terms] OR “female” [All Fields]) OR (“women” [MeSH Terms] OR “women” [All Fields])) AND (“basketball” [MeSH Terms] OR “basketball” [All Fields]) AND ((“monitoring” [MeSH Terms] OR “monitoring” [All Fields]) OR (“training” [MeSH Terms] OR “training” [All Fields]) OR (“internal” [All Fields] OR “physiological” [All Fields])) AND (“load” [MeSH Terms] OR “load” [All Fields]). Furthermore, the reference sections of all the relevant articles were also examined by applying the snowball strategy [13]. The search for published studies was independently performed by 2 different authors (J.E-L. and A.F-V.).

### 2.2. Inclusion and Exclusion Criteria

To select relevant articles, the PICOS model was used to determine the inclusion criteria: P (Population): “female basketball athletes”, I (Intervention): “monitoring internal load practice or competition”, O (Outcome): “physiological response measurements”, and S (study design): “original studies published in journals” [12]. There were no filters applied to the athlete’s physical fitness level, race, or age to increase the power of the analysis. Study participants were categorized into several groups: elite, professional, amateur, and youth players. The elite group was defined by their participation in the Women’s Basketball Association (WNBA), NCAA Division I, Euro League Women and FIBA International Competition. Professional was defined by athletes competing in the first and second divisions in any continent, but were over 19 years old. Amateur level was considered under the level of those mentioned previously. Youth competition was considered for studies in which the participants were all 19 years of age or younger. This clustering has been designed following the previous literature [14].

The systematic bibliographic review included studies that met the following criteria: (1) original investigation, (2) populations were healthy female players at the elite, professional, amateur, or youth levels of any age, (3) articles presented data from monitoring basketball training or competition, including 3v3 and regular 5v5 competition formats, and articles described physiological responses or perceptual measures (internal load) to determine load dose. Friendly games were analyzed jointly with competitive events, while simulated games were analyzed jointly with training events. Only English publications were included. The investigation applied exclusion criteria to experimental protocols, which included: (1) post-event assessments related to recovery or performance status (excluded publications that utilized methods or devices to assess variables before or after the basketball event, such as assessing the loss of neuromuscular function during a jump or the time it took to return to baseline heart rate), (2) validation of research instruments through basketball (the emphasis is on load data to prevent publications that solely compare devices), (3) studies on performance tests in female basketball players (to explore the usage of devices in real situations, during practice and competition), (4) studies on wheelchair basketball, (5) studies with injured participants (injured basketball players may display values that are heterogeneous or differ from the typical responses seen in healthy players), (6) studies for clinical or therapeutic purposes, and (7) doctoral theses, conference oral and poster presentations (primarily focused on peer-reviewed publications of the highest quality).

### 2.3. Study Selection

Two authors (J.E-L. and A.F-V.) identified papers through database searching, and then duplicates were removed. Next, the authors reviewed the titles and abstracts of the remaining publications to determine eligibility for full-text review. All studies that met the inclusion criteria were retrieved and reviewed in full, and any disagreements were resolved by a third reviewer (J.C-G.).

### 2.4. Data Extraction

Once the inclusion/exclusion criteria were applied to each study, the following data were extracted: (1) study source (author/s and year of publication); (2) type of event studied (training or competition); (3) population of the sample, indicating the number of participants, age, and level of activity (elite, professional, semi-professional, amateur, and youth players); (4) observational sample (observation by player and total observation); (5) methods and devices utilized for the quantification of load; (6) variables identified for each method; and (7) outcomes reported for each variable. In the publications in which the variables to be extracted were not shown, information was requested from the corresponding author via e-mail. The final outcomes of the interventions were extracted independently by two authors (J.E-L. and A.F-V.) using a spreadsheet (Microsoft Excel 2019, version 23, Microsoft Inc., Seattle, WA, USA). Subsequently, disagreements were resolved through discussion until a consensus was reached or third-party adjudication (J.C-G.).

### 2.5. Quality Assessment and Risk of Bias

To assess the quality of publications, the Strengthening the Reporting of Observational Studies in Epidemiology (STROBE) statement: guidelines for reporting observational studies was used [15]. Two authors independently assessed the methodological quality and risk of bias (J.E-L. and M.C-M.) of each investigation included for the analysis. Disagreements were resolved by third party evaluation (A.I.).

The following scale was used to classify study quality: (1) good quality (>14 points, low risk of major or minor bias); (2) fair quality (7–4 points, moderate risk of major bias); and (3) poor quality (<7 points, high risk of major bias). The score was obtained through the 22 items of the STROBE checklist. No risk of bias assessment was used because this review was descriptive, and we did not report or discuss effects, associations, or prevalence.

In order to assess inter-rater agreement, Cohen’s Kappa coefficient was calculated for the total scores of the studies based on STROBE.

## 3. Results

### 3.1. Search Strategy

**Figure 1:**
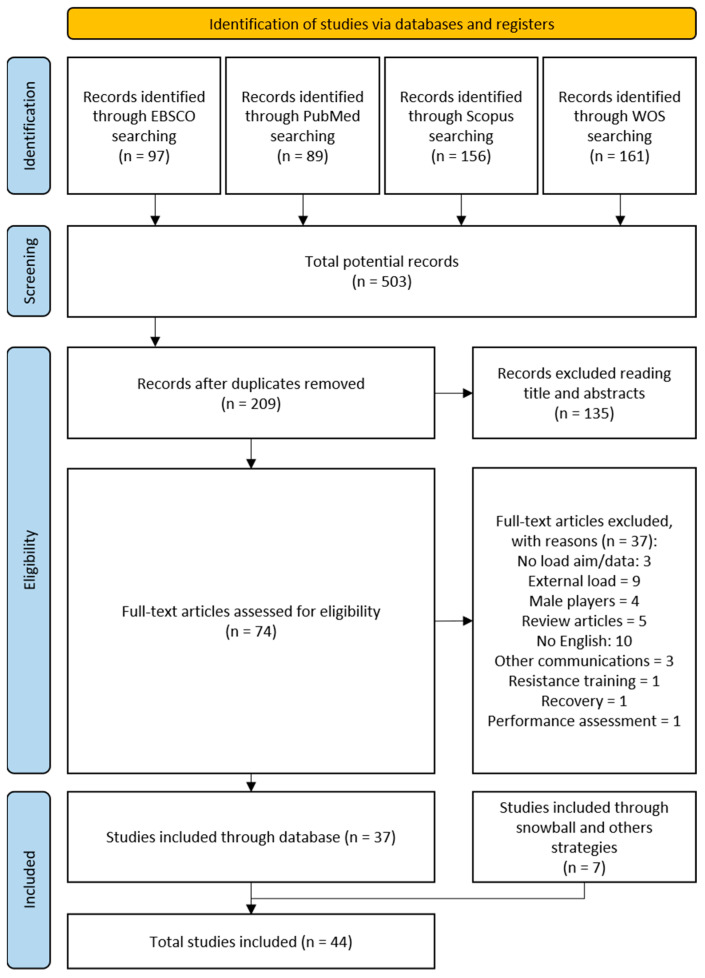
PRISMA diagram.

A selection process was carried out, and a total of 503 potential records were identified through database searches. From these initial 503 articles, 294 duplicates were removed, resulting in a total of 209 publications that underwent title and abstract review. Thereafter, a total of 135 articles were removed after screening the titles and abstracts, leaving 74 articles for full-text assessment of eligibility. Subsequently, a total of 37 articles were eliminated from the review process after full-text peer-reviewed evaluation. The topics and number of studies that were excluded were as follows: three studies were not pursuing the aim to assess the internal load, twelve studies were excluded due to unsuitable outcomes (external load monitoring, resistance training, recovery monitoring, and performance assessment), four studies involving male players were excluded due to inappropriate subjects for inclusion criteria, ten studies were excluded due to non-English language, and five studies were excluded due to unsuitable design (bibliographic review and congress communications). Consequently, 37 studies met the previously defined inclusion criteria and were included in this final systematic review. After a complete snowball search strategy [13], a total of seven more publications were added [16,17,18,19,20,21,22], resulting in a total of forty-four studies included in this systematic review. Figure 1 presents the details of all processes and results obtained by the search strategy.

### 3.2. Populations and Events Studied

Table 1 summarizes the data in relation to the populations and events studied. Regarding the participants’ level of competition, a total of 16 studies included youth athletes, with 12 studies collecting data during training [4,16,20,21,23,24,25,26,27,28,29,30] and 8 studies collecting data during competition [22,26,27,29,30,31,32,33]. Additionally, seven studies investigated the internal load in elite players during training [4,18,27,34,35,36] and six studies investigated the internal load in elite players during competition [26,31,35,36,37,38]. Among the professional players, 13 publications monitored the physiological response, with 7 studies monitoring it during practice [28,39,40,41,42,43,44] and 7 monitoring it during competition [32,39,41,42,45,46,47]. Finally, 12 studies investigated amateur players, with 5 studies monitoring them during practice [48,49,50,51] and 10 studies monitoring them during competition [49,51,52,53,54,55,56]. One of the included publications did not specify the level of competition [57].

Nineteen articles investigated the physiological response of female basketball players only during practice [4,16,20,21,23,25,28,34,39,40,41,43,44,48,50,57,58,59,60], while fourteen publications examined internal load only during competition [17,22,31,33,34,35,37,45,46,52,54,55,61,62] and eleven studies analyzed internal load in both events [24,26,27,29,32,35,36,47,51,53,56]. Among the included studies, 24 reported the type of tasks [4,16,20,21,22,23,24,25,26,27,28,29,34,39,40,41,42,43,44,49,51,53,56,57,58,59,60]. Of the studies that mentioned specific tasks, two publications simulated competition demands during training [24,53]. Regarding competition monitoring, a total of 28 publications recorded data in the 5v5 format [17,18,19,22,24,26,27,29,30,31,32,33,34,35,36,37,38,42,45,46,47,49,51,52,53,54,55,56,61,62], while only 3 studies measured the competition in the 3v3 format [31,37,62], where all were collected during official competition. Among all studies investigating the 5v5 format, there was one friendly match [26].

### 3.3. Subjective Monitoring Load Methods

Table 2 shows all the studies that used subjective methods to monitor internal load. A total of 28 publications used the RPE method as the internal monitoring method [4,16,19,21,22,23,25,26,27,29,31,34,36,37,39,40,41,42,43,44,47,48,50,51,54,56,57,58]. Some authors used the value reported by the players as a metric for analysis. Among these, 19 used this method to measure load in practice [19,21,22,23,25,27,31,35,37,40,43,44,48,50,55,56,57,58], showing a wide data range (2.9–7 AU), while 13 articles described the use of this method in games [19,22,26,27,31,36,37,42,44,47,51,54,56], with a shorter range in both competition formats (5v5: 3.9–6; 3v3: 5.3–5.9 AU). The closed range in the 5v5 format used the 6–20 scale (14.3–15.2 AU).

On the other hand, other authors multiplied this value by the practice or match time [4,16,21,25,26,27,29,34,36,39,41,42,47,50,51,57,58]. One of the most commonly reported metrics was the average session load per player, which exhibited a wide range (253–942 AU). Additional calculations were also observed, such as the team average per session, weekly sum, or average. Finally, few authors described the method or tool used to obtain this subjective value. Notably, those researchers who obtained the value through a mobile app [4,26,42,47], computer [51], or paper and pencil [25] provided some details on the methodology. The timing of data collection was described in a limited number of papers [4,37,44,56].

### 3.4. Sensor-Based Monitoring Load Methods

Table 3 shows all the studies that used sensor-based methods to quantify internal load. The majority of studies included in this systematic review that used sensors to evaluate the physiological response of female basketball players relied on monitoring players’ cardiac response to exercise (27 of 32). With regard to the devices, only four studies provided complete information on the manufacturer, sampling frequency, and body placement (wrist, chest, or upper body) [4,34,35,37]. These sensors were used during practice sessions [4,20,23,28,34,44,48,50,58,60], games [17,19,31,33,35,37,45,46,52,55,61,62], or both events [24,26,30,32,35,47,49,53,57]. Heart rate was the most commonly used method to assess physiological response, although a variety of metrics were obtained and used to measure and evaluate the internal load, including the average HR (HR_Avg_), maximum HR (HR_Max_), minimum HR, percent of maximum HR (%HR_Max_), percent of average HR (%HR_Avg_), total time, or percentage of time spent in different HR zones (2–8 zones), TRIMP_B_, and SHRZ.

During training, the HR_Avg_ exhibited a wider range of values than during competition, with readings ranging from 127.9 to 183.2 beats per minute (bpm) and 144.1 to 145.9 bpm, respectively. In the case of HR_Max_, a similar range was observed between training and competition across different populations, with recorded minimums ranging from approximately 175 to 198 bpm. In addition, the observed %HR_Max_ was slightly lower during training sessions (72.95%) compared to competition (>81.2%). Finally, the use of indices based on HR data was summarized by TRIMP_B_ and SHRZ. For the former, the individual average values (61.7 AU) and team sum values (214–304 AU) were found for each session of practice. Regarding SHRZ, the average range per player and session during training was between 162–352 AU, which was higher than the values during competition (68–80 AU).

A few studies explored other devices to obtain additional information about players’ physiological response to exercise. All these results are summarized in Table 4. Blood lactate analysis was used in four studies during matches, showing a range from 3.2 to 5.7 in different levels of competition [19,31,52], and one study during practice [44], while one study evaluated VO2 during competition, with average values of 33.4 ± 4 mL/kg/min (66.7 ± 7.5% of individual maximal oxygen consumption) [19]. Additionally, one publication employed equations based on heart rate data to estimate calorie consumption during competition [61].

### 3.5. Quality Assessment and Risk of Bias

The publications in this review were classified into three categories based on their STROBE score. Of the total 44 studies, 28 were considered good quality, while 16 were fair. Table 1 displays the STROBE score and qualification of each study. The resulting coefficient was 0.863, indicating substantial agreement between rater 1 and rater 2. The significance level was below <0.001. In addition, only 0.51% (5 out of 968) of items were rated differently by the two reviewers, and the third reviewer’s criteria were used to resolve any discrepancies.

**Table 1:** Basic characteristics of publications.

Publication	*n*	Level	Age	Event Registered	Method	Study Quality (Rate)
Anderson et al. (2003) [16]	12	Y	18–22	P	S	Good (15)
Matthew et al. (2009) [52]	9	A	25.8 ± 2.5	G	DB	Fair (14)
Narazaki et al. (2009) [19]	6	E	20.0 ± 1.3	P	S; DB	Fair (14)
Delextrat et al. (2012) [39]	9	P	24.3 ± 4.1	P; G	S	Fair (14)
Klusemann et al. (2012) [23]	8	Y	17.4 ± 0.7	P	S; DB	Good (16)
Scanlan et al. (2012) [17]	12	A	22.0 ± 3.7	G	DB	Good (15)
Atli et al. (2013) [20]	12	Y	15.5 ± 0.5	P	DB	Good (15)
Azpiroz et al. (2013) [22]	87	Y	U12	G	S	Fair (13)
Nunes et al. (2014) [58]	19	E	26.0 ± 5.0	P	S; DB	Good (16)
Abad et al. (2016) [24]	15	Y	16.9 ± 1.1	P	DB	Fair (12)
Vencúrik et al. (2016) [45]	10	Pro	20.4 ± 2.8	G	DB	Fair (10)
Legg et al. (2017) [21]	10	Y	18 ± 2	P	S	Fair (14)
Messias et al. (2017) [57]	8	NR	20 ± 1	P	S	Fair (12)
Vallés Ortega (2017) [40]	12	P	21.9 ± 4.8	P	S	Fair (13)
Vallés Ortega et al. (2017) [54]	12	A	17.1 ± 0.7	G	S	Fair (13)
Batalla et al. (2018) [55]	10	A	21.3 ± 2.7	G	DB	Fair (14)
Cruz et al. (2018) [41]	10	P	17.2 ± 0.4	P	S	Good (15)
Montgomery et al. (2018) [31]	208	E; Y	22.9 ± 5.6	G	S; DB	Good (16)
Sánchez et al. (2018) [59]	6	A	14.3 ± 0.5	P	S; DB	Fair (13)
Sanders et al. (2018) [35]	10	E	19.8 ± 1.3	G	DB	Good (16)
Coyne et al. (2019) [18]	12	E	27.8 ± 3.6	P	S	Good (17)
Lupo et al. (2019) [34]	15	E	16.7 ± 0.5	P	S	Good (15)
Paulauskas et al. (2019) [42]	29	Pro	21.0 ± 5.0	P; G	S	Good (17)
Reina et al. (2019) [49]	10	A	21.7 ± 3.7	P; G	DB	Good (18)
Reina et al. (2019) [30]	12	Y	U13	P; G	DB	Good (15)
Sanders et al. (2019) [35]	13	E	19.6 ± 1.3	P; G	DB	Good (15)
Vala et al. (2019) [46]	17	Pro	23.4 ± 2.1	G	DB	Fair (12)
Kraft et al. (2020) [50]	-	A	-	P	S	Fair (14)
Lastella et al. (2020) [25]	11	Y	17.3 ± 0.9	P	S	Good (16)
Lukonaitene et al. (2020) [26]	24	E; Y	18.8 ± 0.7	P; G	S; DB	Good (15)
Otaegi et al. (2020) [27]	19	Y	16.1 ± 0.7	P; G	S	Good (15)
Sansone et al. (2020) [51]	11	A	22.0 ± 3.0	P; G	S	Good (17)
Stauton et al. (2020) [43]	9	Pro	26 ± 3	P	S	Good (15)
Suárez-Iglesias et al. (2020) [28]	10	Pro; Y	18.6 ± 3.5	P	DB	Good (15)
Adrianova et al. (2021) [61]	10	Pro	183.9 ± 8.7	G	DB	Fair (10)
Brini et al. (2021) [44]	12	Pro	24.8 ± 1.8	P	S; DB	Good (16)
Coyne et al. (2021) [36]	13	E	29.0 ± 3.7	P; G	S	Good (18)
Espasa-Labrador et al. (2021) [4]	13	E; Y	16.3 ± 1	P	S; DB	Good (16)
Piñar et al. (2021) [47]	13	Pro	25.2 ± 7.3	G	S; DB	Good (17)
Senbel et al. (2021) [29]	NR	Y	NR	P; G	S	Fair (11)
Vencúrik et al. (2021) [32]	18	Pro; Y	18.8 ± 1.9	G	DB	Good (19)
Batalla-Gavalda et al. (2022) [56]	10	A	21.3 ± 2.7	G	S; DB	Good (18)
Gutiérrez-Vargas et al. (2022) [33]	32	Y	16.2 ± 1	G	DB	Good (15)
Willberg et al. (2022) [39]	37	E	23.5 ± 4.1	G	S; DB	Good (15)

NR: not reported; A: amateur; Y: youth; Pro: professional; E: elite; P: practice; G: game; S: subjective methods; DB: device-based monitoring methods.

**Table 2:** Internal load monitoring subjective perception-based method.

Publication (*n*; Level; Age)	Event		Observation		Method	Metrics	Tool(s) and Methodology	Outcome
	Practice Game	Study-Defined Mode(s)	Obs. by Player	Statistical Units				
Anderson et al. (2003) [16] (12; Y; A; 18–22)	P	NR	NR	NR	RPE (1–10)	sRPE	NR	NR
Narazaki et al. (2009) [19] (6; E; 20.0 ± 1.3)	G	5v5 OG	6	36	RPE (6–20)	RPE	NR	Player’s average: 14.3 ± 1.9
Delextrat et al. (2012) [39] (9; Pro; 24.3 ± 4.1)	P	FCS, SSG, DT, TT	5	45	RPE (1–10)	sRPE	NR	NR
Klusemann et al. (2012) [23] (8; Y; 17.4 ± 0.7)	P	SSG (2v2; 4v4)	19	152	RPE (1–10)	RPE	NR	Player’s average by task format: 4v4; 2v2; Half court; Full court; 2 × 5 min; 4 × 2.5 min 6 ± 2; 8 ± 2; 6 ± 2; 7 ± 2; 7 ± 2; 7 ± 2
Azpiroz et al. (2013) [22] (87; Y; U12; 16.9 ± 1.1)	G	5v5 OG	NR	NR	RPE (1–10)	RPE	NR	Player’s average: 4.48 ± 1.65
Nunes et al. (2014) [58] (19; E; 26 ± 5)	P	FCS	NR	NR	RPE (1–10)	RPE; sRPE	NR	Player’s average: RPE: 3.9 ± 1.5 sRPE: 321 ± 127
Legg et al. (2017) [21] (10; Y; 18 ± 2)	P	NR	NR	NR	RPE (1–10)	sRPE	NR	Player’s average values by moment of season: Pre-season: 3195 ± 1083 Mid-season: 4344 ± 1376
Messias et al. (2017) [57] (8; A; 20 ± 1)	P	TaT; TeT	42	336	RPE (1–10)	RPE; sRPE	NR	Weekly team’s average: RPE: 3.9 ± 0.9 sRPE: 413 ± 163.8
Vallés Ortega (2017) [40] (12; Pro; 21.91 ± 4.81)	P	FCS	NR	NR	RPE (1–10)	RPE	NR	Team’s average: 3.12 ± 0.54
Vallés Ortega et al. (2017) [54] (12; A; 17.08 ± 0.67)	G	5v5 OG	6	50	RPE (1–10)	RPE	NR	Team’s average: 4.16 ± 1.05
Cruz et al. (2018) [41] (10; Pro; 17.2 ± 0.4)	P	FCS	NR	NR	RPE (1–10)	sRPE	NR	Weekly team’s sum: 1584.3 ± 237.4
Montgomery et al. (2018) [31] (208; E, Y; 22.9 ± 5.6)	G	3v3 OG	NR	635	RPE (1–10)	RPE	NR	Player’s average by competition: Wch; ECh; U18 RPE: 5.3 ± 0.3; 5.8 ± 0.6; 5.9 ± 0.6
Sánchez et al. (2018) [59] (6; A; 14.3 ± 0.5)	P	SSG	2	12	RPE (1–10)	RPE	NR	Player’s average: 5.80 ± 1.23
Coyne et al. (2019) [18] (13; E; 29.0 ± 3.7)	P; G	P: NR G: 5v5 OG	126.3	1642	RPE (1–10)	RPE; sRPE	NR	RPE average: 5.53 ± 1.67 RPE average in practice: 5.37 ± 1.62 Weekly load: 4588 ± 1587 Games data: RPE average 5.53 ± 1.67 RPE average in competition: 7.11 ± 1.22 Weekly load: 4588 ± 1587
Lupo et al. (2019) [34] (15; E; 16.7 ± 0.5)	P	FCS	19	268	RPE (1–10)	sRPE	NR	Player’s average by session: strength; conditioning; technique sRPE: 521 ± 25.6; 555 ± 34.8; 514 ± 20.5
Paulauskas et al. (2019) [42] (29; Pro; 21 ± 5)	P; G	P: FCS G: 5v5 OG	96–144	2784–4176	RPE (1–10)	sRPE	Personal mobile device using Cloud-based software (Google Forms, Menlo Park, CA, USA)	Weekly sRPE player’s average: 1722 ± 715 Weekly sRPE player’s average during game clustered: Low playing time group: 720.3 ± 200.9 High playing time group: 903.1 ± 208.9
Kraft et al. (2020) [50] (NR; NR; NR)	P	NR	NR	124	RPE (1–10)	RPE; sRPE	NR	Player’s average: RPE: 5.1 ± 1.8 SRPE: 711 ± 282
Lastella et al. (2020) [25] (11; Y; 17.3 ± 0.9)	P	FCS	111	1221	RPE (1–10)	sRPE	Paper and pencil	Session’s average clustered by type: LTLS: 274 ± 136 MTLS: 576 ± 221 HTLS: 1186 ± 309
Lukonaitene et al. (2020) [26] (24; E, Y; 18.8 ± 0.7)	P; G	P: FCS G: 5v5 FG	33	792	RPE (1–10)	sRPE	Personal mobile device using Cloud-based software (Google Forms, Menlo Park, CA, USA)	Data include practice and game average Team’s average: U20; U18 sRPE: 617.29 ± 328.24; 942.82 ± 436.51
Otaegi et al. (2020) [27] (19; Y; 15 ± 0.7)	P; G	P: FCS G: 5v5 OG	50	478	RPE (1–10)	RPE; sRPE	Ask personally by coach	Team’s average by teams: U15; U16 Daily RPE: 2.9 ± 0.3; 3.1 ± 0.6 Daily sRPE: 253 ± 27; 259 ± 50 Week sum sRPE: 10.9 ± 1.9; 13.9 ± 3.0 Week sum sRPE: 879 ± 140; 1073 ± 260 Games data: RPE (U15; U16): 3.6 ± 1.2; 4.5 ± 1.0 sRPE (U15; U16): 316 ± 115; 378 ± 96
Sansone et al. (2020) [51] (11; A; 22.0 ± 3.0)	P; G	P: FCS G: 5v5 OG	40	40	RPE (1–10)	sRPE	Registered individually with laptop	Player’s average during practice: 428 ± 114 Weekly sRPE player’s average: 1561 ± 177 NR data during games
Stauton et al. (2020) [43] (9; Pro; 26 ± 3)	P	WU; SD; OD; DD; MS	NR	NR	RPE (1–10)	RPE	NR	Player’s average by type of task: WU; SD; OD; DD; MS 4.8 ± 0.1; 6.5 ± 0.2; 6.0 ± 0.1; 7.4 ± 0.0; 7.4 ± 0.0 Player’s average by session: 6.42 ± 0.1
Brini et al. (2021) [44] (12; Pro; 24.8 ± 1.8)	P	SSG	NR	NR	RPE (1–10)	RPE	RPE: after each SSG and 30 after practice, NR tool	Player’s average: 7.0 ± 0.8
Coyne et al. (2021) [36] (13; E; 29.0 ± 3.7)	P; G	FCS G: 5v5 OG	NR	NR	RPE (1–10)	sRPE	RPE: 30′ after event; NR	Daily average: 648 ± 496 Weekly average (including practice and game): 4588 ± 1597
Espasa-Labrador et al. (2021) [4] (13; E, Y; 16.3 ± 1)	P [39]	FCS	35	164	RPE (1–10)	sRPE	Quanter Mobile App (Kvantia, Helsinki, Finland)	Average per session: sRPE: 765.3 ± 174.9; SHRZ: 276.1 ± 61.9; TRIMPB: 61.7 ± 10.1
Piñar et al. (2021) [37] 13; Pro; 25.2 ± 7.3	P; G	P: NR G: 5v5 OG	28	NR	RPE (1–10)	sRPE	Quanter Mobile App (Kvantia, Helsinki, Finland)	Weekly load sRPE (including practice events) Pre-season: 2168 ± 911 First round: 1612 ± 881 Second round: 1750 ± 729
Senbel et al. (2021) [29] (NR; NR; NR)	P; G	FCS, RT, CT	NR	NR	RPE (1–10)	RPE; sRPE	NR	NR
Batalla-Gavalda et al. (2022) [56] (10; A; 21.3 ± 2.71)	P; G	P: FCS G: 5v5 OG	NR	P: NR G: 68	RPE (6–20)	RPE	RPE: 30′ after game and 10′ after practice. Reported individually in an isolated area	Data of 10 games (min; average; max) RPE: 15.2 ± 2.4; 16.8 ± 1.8; 18 ± 1.1
Willberg et al. (2022) [37] (37; Pro; 23.5 ± 4.1)	G	5v5 OG 3v3 OG	NR	NR	RPE (1–10)	RPE	RPE: 15–30′ after game	Team’s average: 5v5 OG: 6 ± 2 3v3 OG: NR

NR: not reported; A: amateur; Y: youth; Pro: professional; E: elite; P: practice; G: game; SSG: small side game; 2v2: two versus two players; 4v4: four versus four players; 5v5: five versus five players; FC: full-court task; HC: half-court task; DT: defensive task; SG: simulated game conditions; WD: without defense; ST: superiority (offense) task; RT: resistance training; CT: conditioning tasks; TT: total time; UT: useful time; LTLS: low training load session; MLTS: moderate load training load session; HLTS: high load training session; RPE: rating perceived exertion (AU); sRPE: session-rating perceived exertion (AU); U(15,16,18): under age group; AU: arbitrary units.

**Table 3:** Internal load HR sensor-based.

Publication (n; Level; Age)	Event		Observation		Method	Metrics	Tool(s); SF; Body Place Worn	Outcome
	Practice Game	Study-Defined Practice Mode(s)	Obs. by Player	Total Statistical Units				
Matthew et al. (2009) [52] (9; A; 25.8 ± 2.5)	G	5v5 OG	9	81	HR	% of time spent >85% HRMax; HRAvg	Polar S810 (Polar Electro Oy, Kempele, Finland); 15-s SF; NR	Mean 80.4% time at HR greater than 85% of HRMax (relative to total time) Mean 93.1% time at HR greater than 85% of HRMax (relative to live time) Mean 166.3 ± 9.4 HRAvg in 1st half and 163.3 ± 9.0 in 2nd half.
Narazaki et al. (2009) [19] (6; E; 20.0 ± 1.3)	G	5v5 OG	6	36	HR	HRPlay; HRRest	Polar watch (Polar Electro Oy, Kempele, Finland); NR SF; wrist	HRPlay (bpm): 168.7 ± 11.0 HRRest (bpm): 152.5 ± 11.5
Klusemann et al. (2012) [23] (8; Y; 17.4 ± 0.7)	P	SSG (2v2; 4v4)	19	152	HR	%HRMax, %HRAvg, % time spent in two different HR zones	Suunto Heart Rate sensor (Suunto™, Vantaa, Finland); NR; NR	Player’s average by task format: 4v4; 2v2; HC; FC; 2 × 5 min; 4 × 2.5 min % HRMax: 92 ± 3; 92 ± 3; 92 ± 3; 92 ± 3; 92 ± 3; 92 ± 2 % HRAvg: 83 ± 5; 86 ± 4; 84 ± 5; 85 ± 4; 86 ± 4; 83 ± 3 % time in Z4: 51 ± 20; 55 ± 24; 46 ± 27; 56 ± 19; 53 ± 26; 58 ± 9 % time in Z5: 22 ± 25; 30 ± 31; 20 ± 27; 25 ± 27; 33 ± 32; 14 ± 13
Scanlan et al. (2012) [17] (10; A; 21.7 ± 3.65)	G	5v5 OG	8	NR	HR	HRAvg; %HRMax	Polar Team System (Polar Electro, Oy, Kempele, Finland); 5-s SF; NR	Team’s average by quarters: HRAvg; %HRMax Q1: 165 ± 4; 83.2 ± 2.6 Q2: 163 ± 5; 84 ± 2.6 Q3: 161 ± 4; 81.3 ± 1.9 Q4: 162 ± 6; 81.5 ± 2.9 1st Half: 163 ± 3; 82.4 2nd Half: 161 ± 4; 81.2 ± 1.9 Match: 162 ± 3; 82.4 ± 1.3
Atli et al. (2013) [20] (12; Y; 15.5 ± 0.5)	P	HC, FCS	NR	NR	HR	HRAvg, %HRMax	Polar S810 HR (Polar Electro, Oy, Kempele, Finland); 5-s SF; NR	Player’s average values by type of task: HC; FC HRAvg: 161.8 ± 6.2; 180.9 ± 5.7 %HRMax: 76.3 ± 2.5; 85.6 ± 3.1
Nunes et al. (2014) [58] (19; E; 26 ± 5)	P	FCS	NR	NR	HR	SHRZ	NR	Player’s average: 255 ± 62
Abad et al. (2016) [24] (15; Y; 16.9 ± 1.1)	P; G	P: 5v5 SG G: 5v5 OG	1	15	HR	HRMax	Polar Team Pro (Polar, Kempele, Finland); NR; NR	Practice and game: HRmax: 195.27 ± 8.40
Vencúrik et al. (2016) [45] (10; Pro; 20.4 ± 2.8)	G	5v5 OG	1	10	HR	%HRMax, time spent in five different HR zones	Suunto Team Pack (Suunto Oy, Vantaa, Finland); 2-s SF; NR	Player’s average by position (point guards; forwards; centers): %HRmax: 88.2 ± 3.5; 87.8 ± 3.1; 88.9 ± 3.4 Z3 (<85%): 24.0 ± 19.4; 24.3 ± 12.5; 19.8 ± 13 Z4 (85–95%): 63.7 ± 17.6; 67.9 ± 10.7; 65.9 ± 15.8 Z5 (>95%): 12.3 ± 13.9; 7.9 ± 10.8; 14.2 ± 16.2
Batalla et al. (2018) [55] (10; A; 21.3 ± 2.71)	G	5v5 OG	10	100	HR	%HRMax	Suunto Team Pack (Suunto Oy, Vantaa, Finland); NR; NR	%HRMax by quarters: Q1: 90.2 ± 4.4; Q2: 90.3 ± 4.2 Q3: 89.6 ± 3.4; Q4: 90.4 ± 2.5
Montgomery et al. (2018) [31] (208; E, Y; 22.9 ± 5.6)	G	3v3 OG	NR	635	HR	HRMax; HRAvg	Polar T34 (Polar, Kemple, Finland); NR; NR	HRMax: 198 ± 9 HRAvg: 165 ± 18
Sanders et al. (2018) [35] (10; E; 19.8 ± 1.3)	G	5v5 OG	31	310	HR	HRMax; HRAvg; time spent in six different HR zones; SHRZ	Polar Team Pro (Polar, Kempele, Finland); NR; NR	Average by position (guards; forwards; centers): HRmax: 195.7 ± 6.7; 187.3 ± 8.8; 194.2 ± 8.8 HRavg: 146.0 ± 15.1; 149.9 ± 14.5; 151.1 ± 14.0 Z1 (50–60%): 4.3 ± 2.8; 3.0 ± 3.5; 3.6 ± 3.9 Z2 (60–70%): 3.2 ± 2.0; 3.4 ± 2.1; 4.7 ± 3.1 Z3 (70–76%): 1.4 ± 0.8; 2.1 ± 1.4; 2.6 ± 2.0 Z4 (77–84%): 1.9 ± 1.1; 3.5 ± 2.0; 2.5 ± 1.5 Z5 (85–100%): 9.2 ± 3.8; 10.0 ± 4.3; 8.4 ± 3.5 85% HRmax: 61.1; 69.2; 66.3 SHRZ: 68.3 ± 15.1; 80.1 ± 23.1; 72.9 ± 21.2
Lupo et al. (2019) [34] (15; E; 16.7 ± 0.5)	P	FCS	19	268	HR	SHRZ	Polar H7 (Polar Electro Oy, Kepele, Finland); 1-s SF; chest	Player’s average by type of session: Strength: 229 ± 14.4 Conditioning: 229 ± 19 Technique: 162 ± 12.1
Reina et al. (2019) [49] (10; A; 21.7 ± 3.65)	P; G	P: SG G: 5v5 OG	47	155	HR	HRMax, HRAvg, %HRMax, time spent in six different HR zones	Garmin™; NR; NR	Team’s average: HRMax: 175.18; HRAvg: 145.91; %HRMax: 72.95; Z1 (50–60%): 17.78; Z2 (60–70%): 19.32; Z3 (70–80%): 23.28; Z4 (80–90%): 27.38; Z5 (90–95%): 9.19; Z6 (>95%): 1.27 Average in games: Average team values HRMax: 192.33; HRAvg: 169.18; %HRMax: 84.59; Z1 (50–60%): 3.66; Z2 (60–70%): 6.30; Z3 (70–80%): 12.35; Z4 (80–90%): 37.14; Z5 (90–95%): 31.84; Z6 (>95%): 8.09
Reina et al. (2019) [30] (12; Y; U13)	P; G	P: NR G: 5v5 OG	35	420	HR	%HRAvg, %HRMax	Garmin™; NR; NR	NR
Sanders et al. (2019) [35] (13; E, Y; 19.6 ± 1.3)	P; G	P: NR G: 5v5 OG	NR	NR	HR	HR, SHRZ	Polar Team Pro (Polar, Kempele, Finland); 1-s SF; chest	Showed data including practice and game average Average values for groups (large; moderate; minimal): HRmax: 196.5 ± 1.4; 195.5 ± 1.8; 193.2 ± 1.6 HRavg: 132.6 ± 1.1; 130.8 ± 1.0; 127.9 ± 1.6 SHRZ: 352.2 ± 11.6; 314.5 ± 13.4; 276.5 ± 13.2 Time >85% HRmax (min): 21.6 ± 1.2; 20.0 ± 1.4; 16.4 ± 1.6
Vala et al. (2019) [46] (17; Pro; 23.4 ± 2.1)	G	5v5 OG	NR	16	HR	HRAvg, %HRMax; time spent in five different HR zones	Polar Team System 2 (Polar, Kemple, Finland); NR; NR	HRAvg by league and position: 1st league; 2nd league Guards: 174.8 ± 9.2; 183.3 ± 6.7 Forwards: 182.9 ± 12.3; 169.7 ± 6.7 Centers: 190.6 ± 11.3; 174.4 ± 9.1 Total: 183.2 ± 12.8; 176.1 ± 10.3 Average % HRmax by league and position: 1st league; 2nd league Guards: 91.1 ± 5.6; 90.1 ± 4.4 Forwards: 92.3 ± 5.6; 85.7 ± 3.4 Centers: 92.2 ± 4.8; 90.3 ± 2.9 Total: 91.9 ± 5.3; 88.8 ± 4.2 % time spent by positions: guards; forwards; centers Z1 (<80%): 0.64; 0.00; 0.00 Z2 (80–85%): 7.40; 3.67; 3.90 Z3 (85–90%): 42.41; 27.34; 17.36 Z4 (90–95%): 40.42; 45.5; 57.82 Z5 (>95%): 9.13; 23.49; 20.93
Kraft et al. (2020) [50] (NR; NR; NR)	P	NR	NR	124	HR	HR	Polar H7 sensor and Polar Team System (Polar, Kemple, Finland); NR; NR	Player’s average values: SHRZ: 313 ± 112
Lukonaitene et al. (2020) [26] (24; E, Y; 18.8 ± 0.7)	P; G	P: FCS G: 5v5 FG	33	792	HR	TRIMPB	H10 Polar Sensor (Polar, Kemple, Finland); NR; NR	Team’s average U20: 214.60 ± 109.42 U18: 304.95 ± 171.83 Showed data including practice and game
Suárez-Iglesias et al. (2020) [28] (10; Pro; 18.6 ± 3.5)	P	1v1; DT	12	120	HR	%HRMax; %HRAvg; %Time spent 80–89% HRMax; %Time spent 90–100% HRMax; SHRZ	Suunto Team Pack (Suunto Oy, Vantaa, Finland); 5-s SF; NR	Team’s average by tasks (1v1; defense): %HRMax: 93.3 ± 4.9; 94.1 ± 5.6 %HRAvg: 83.6 ± 6.3; 85.1 ± 6.5 %Time spent at 80–89% HRMax: 43.7 ± 20.2; 40. ± 23.8 %Time spent at 90–100% HRMax: 25.7 ± 29.3; 45.2 ± 31.7 SHRZ: 3.8 ± 0.6; 4.3 ± 0.5
Adrianova et al. (2021) [61] (10; Pro; 23 ± 3)	G	5v5 OG	89	NR	HR	HRMax, HRAvg, number of kcal	Polar Team System HR sensors H10 (Polar, Kemple, Finland); NR; NR	Player’s average by season: season 2018/19; season 2019/20 HRMax: 197; 187 HRAvg: 137.7; 140.3 Total kcal: 875.7; 972.6 Kcal/min: 41.6; 46.9
Brini et al. (2021) [44] (12; Pro; 24.8 ± 1.8)	P	SSG	NR	NR	HR	HRAvg	Polar Team System (Polar, Kemple, Finland); 5-s SF; NR	HRAvg: 187.1.7
Espasa-Labrador et al. (2021) [4] (13; E, Y; 16.3 ± 1)	P	FCS	35	164	HR	SHRZ; TRIMP;	Polar Team Pro System (Polar, Kemple, Finland); 200Hz SF; chest	Player’s average during session: SHRZ: 276.1 ± 61.9 TRIMPB: 61.7 ± 10.1
Piñar et al. (2021) [47] (13; Pro; 25.2 ± 7.3)	P; G	P: NR G: 5v5 OG	28	NR	HR	NR	M400 Polar (Polar, Kemple, Finland); NR; NR	NR
Vencúrik et al. (2021) [32] (18; Y, Pro; 18.8 ± 1.9)	P; G	P: NR G: 5v5 OG	14	122	HR	% of time spend in three different HR zones	Suunto Team; Pack telemetry system (Suunto Oy, Vantaa, Finland); 2-s; NR	NR
Batalla-Gavalda et al. (2022) [56] (10; A; 21.3 ± 2.71)	P; G	P: FCS G: 5v5 OG	NR	P: NR G: 68	HR	HRAvg	Suunto Team Pack (Suunto Oy, Vantaa, Finland); NR; NR	Player’s average during 10 games: HRMin: 125.2 ± 10.9 HRAvg: 140.4 ± 11.1 HRMax: 147.3 ± 10.6
Gutiérrez-Vargas et al. (2022) [33] (32; E, Y; 16.2 ± 1)	G	5v5 OG	NR	NR	HR	HRMax, % time spent in five different HR zones	Garmin™; NR; NR	Average values by position: team; guards; forwards; centers Winning game: HRMax: 188.3 ± 17.3; 188 ± 23.6; 188 ± 17.7; 189 ± 10.6 50–60% HR: 6.7 ± 14.1; 6 ± 16; ±7 ± 13.8 60–70% HR: 9.1 ± 11.3; 12.8 ± 13.2; 6.4 ± 7.5; 8.1 ± 13.1 70–80% HR: 15.8 ± 13.6; 21 ± 17.9; 16.5 ± 14.2; 10 ± 8.7 80–90% HR: 30.9 ± 17.8; 32 ± 21.2; 31.5 ± 15.9; 21.1 ± 16.4 >90% HR: 21.2 ± 15.9; 16.4 ± 17.4; 24.2 ± 15.5; 28.9 ± 14.9 Losing game: HRMax: 189.2 ± 16.2; 189.5 ± 20.7; 188.7 ± 14; 189.6 ± 16.1; 50–60% HR: 8.4 ± 19.2; 12.2 ± 22.6; 7.5 ± 19.0; 5.6 ± 16.1; 60–70% HR: 8.25 ± 12.4; 8.2 ± 10.6; 7.4 ± 9.2; 9.1 ± 17.2; 70–80% HR: 11.9 ± 10.5; 12.1 ± 10.7; 13.9 ± 11.9; 9.8 ± 9; 80–90% HR: 29.9 ± 17.8; 29.3 ± 17.3; 32.1 ± 17.7; 28.1 ± 18.5 >90% HR: 24.4 ± 16; 22.4 ± 15.5; 23.7 ± 16.2; 27 ± 16.3
Willberg et al. (2022) [37] (37; Pro; 23.5 ± 4.1)	G	5v5 OG 3v3 OG	NR	NR	HR	HR_Max_, HR_Avg_, time spent in eight different HR zones	Vector Elite Vest (Catapult Sports, Melbourne, Australia); 10 Hz; Upper body	Team’s average by type of competition: HRAvg; Dominant HR Zone 5v5 OG: 6 ± 2; 151.4 ± 22.7; 160–180 (zone 7) 3v3 OG: NR; 160.8 ± 16.1; 160–180 (zone 7)

NR: not reported; A: amateur; E: elite; Y: youth; Pro: professional; G: game; P: practice; 5v5: five versus five players; 4v4: four versus four players; 3v3: three versus three players; 2v2: two versus two players; 1v1: two versus two players; SSG: small side game; FC: full-court task; HC: half-court task; ST: superiority (offense) task; WD: without defense; DT: defensive task; SG: simulated game conditions; HR: heart rate (beats per minute); HR_Max:_ maximal heart rate (beats per minute); HR_Avg_: average heart rate (beats per minute); HR_Min_: minimal heart rate (beats per minute); HR_Play_: heart rate playing (beats per minute); HR_Rest_: heart rate resting (beats per minute); >85%HR_Max_: time spent over 85% of individual maximal heart rate; SHRZ: summated heart rate zones; TRIMP_B_: Banister’s training impulses; U(13,18,20): under age group; SF: sampling frequency; Z1: time spent in zone 1 of heart rate; Z2: time spent in zone 2 of heart rate; Z3: time spent in zone 3 of heart rate; Z4: time spent in zone 4 of heart rate; Z5: time spent in zone 5 of heart rate; Z6: time spent in zone 6 of heart rate; kcal: kilo calories; Q1: 1st quarter; Q2: 2nd quarter; Q3: 3rdquarter; Q4: 4th quarter; AU: arbitrary units.

**Table 4:** Other devices used to monitor the load.

Publication (n; Level; Age)	Event		Observation		Method	Metrics	Tool(s); Characteristics	Outcome
	Practice Game	Study-Defined Practice Mode(s)	Obs. by Player	Total Statistical Units				
Matthew et al. (2009) [52] (9; A; 25.8 ± 2.5)	G	5v5 OG	9	81	BLC	mmol·L^−1^	Analox LM5 (Analox Instruments Ltd., London, UK)	Player’s average: 1st Half: 5.4 ± 1.5; 2nd Half: 5.0 ± 1.4 Game: 5.2 ± 2.7 (55.9% of maximum)
Narazaki et al. (2009) [19] (6; E; 20.0 ± 1.3)	G	5v5 OG	6	36	BLC; VO_2_	mmol·L^−1^; ml/Kg/min, %VO_2Max_	NR, Portable VO2000 (Medical Graphics Corp., St. Paul, MN, USA); VO_2_: 0.05 Hz	Player’s average: BLC_Play_: 3.2 ± 0.9 VO_2Play_ (ml/Kg/min): 33.4 ± 4.0 VO_2Play_ (%VO_2Max_): 66.7 ± 7.5 VO_2Rest_ (ml/Kg/min): 21.3 ± 2.1 VO_2Rest_ (%VO_2Max_): 42.7 ± 6.1
Scanlan et al. (2012) [17] (10; A; 21.7 ± 3.65)	G	5v5 OG	8	NR	BLC	mmol·L^−1^	Accusport Lactate Analyser (Boehringer, Mannheim, Germany)	Team’s average by different periods: Q1: 3.6 ± 0.7; Q2: 4.6 ± 2.4; Q3: 3.4 ± 0.6; Q4: 3.5 ± 1.2 1st Half: 4.1 ± 1.7; 2nd Half: 3.4 ± 1.0 Game: 3.7 ± 1.4
Montgomery et al. (2018) [31] (208; E, Y; 22.9 ± 5.6)	G	3v3 OG	NR	635	BLC	mmol·L^−1^	Lactate Scout+ (SensLab GmbH, Germany)	Player’s average by competition: WCh: 5.98 ± 0.98 ECh: 5.55 ± 0.50 U18: 5.69 ± 0.62
Brini et al. (2021) [44] (12; Pro; 24.8 ± 1.8)	P	SSG	NR	NR	BLC	mmol·L^−1^	3 min after practice. Lactate Pro, Arkray, Japan	NR

NR: not reported; A: amateur; E: elite; Y: youth; Pro: professional; P: practice; G: game; 5v5: five versus five players; 3v3: five versus five players; SSG: small side game; BLC: blood lactate concentration; VO_2_: oxygen consumption; VO_2Max_; maximal oxygen consumption; WCh: World Championship; ECh: European Championship; U18: under 18 years old.

## 4. Discussion

The main finding of this systematic review was the quantification of internal load in female basketball players, which was most commonly achieved using methods based on RPE and HR metrics. These measures were then incorporated into various equations to evaluate the physiological response to activity. Some publications also incorporated the use of BLC and VO_2_ measurements. However, the use of multiple metrics to quantify physiological responses, often with little standardization, led to significant heterogeneity among the studies. This heterogeneity may limit the ability to draw meaningful comparisons between the studies. This review provides a reference framework that highlights the methods and metrics used in current research on female basketball by comparing the extracted data obtained in all included works. The most relevant findings are discussed in detail below.

### 4.1. Subjective Methods for Internal Load Monitoring

The RPE has emerged as a widely used method for load monitoring in several team sports [5], including basketball [7]. This method has two major advantages over other techniques: it is (1) cost-effectiveness and (2) non-invasive. Such advantages address two common challenges experienced by many women’s basketball teams, namely the restrictions prohibiting players from using sensors during matches and the limited financial resources to access sensor-based monitoring techniques. As a result, RPE has been frequently employed in studies evaluating internal load during basketball matches, as evidenced in this systematic review (14/29 of publications reporting its use).

Although the RPE method offers benefits, it also poses certain limitations. Despite the fact that most of the studies referenced Borg’s (1970) work to justify the scale employed [63], few studies addressed the methodological aspects associated with the RPE method. Halperin et al. (2019) underscore that the method requires (1) identifying the construct intended to be obtained from the response (fatigue, discomfort, or intensity/hardness), (2) establishing the temporal range evaluated by the athlete (full session or specific tasks), (3) providing researchers and athletes with instructions (precise question, timing, verbal communication, etc.) and employing an appropriate scale (qualitative visual or quantitative verbal: between 0–10, 6–20, etc.), and (4) evaluating the entire body or specific parts [64]. These methodological aspects may account for the heterogeneity of the data found, particularly in the assessment of practice. Moreover, the athletes’ experience with the method must also be considered. This need for familiarization is evident when observing data in young athletes (U15 and U16), who report the lowest training values (2.9–3.1 AU) [27]. All the above-described aspects modify the athlete’s response, and few studies detailed them in their methods section. Some authors note the time elapsed between the end of the event and the question, which ranges from 0 to 30 min [37,44,56], the instrument employed to gather responses (paper and pen, a computer, or mobile applications), and whether the assessment was conducted individually and in a designated area. These methodological processes, albeit good, may be insufficient, and thoughtful consideration of the aforementioned aspects is crucial for appropriate load monitoring and management.

In addition to the limitations of RPE that we noted above, a recent systematic review with meta-analysis examined the subjective perception of effort in female athletes throughout the menstrual cycle [65]. Although the authors found that hormonal variations throughout the different phases of the menstrual cycle could affect certain evaluated items, such as motivation, competitiveness, sleep, muscle soreness, and fatigue, RPE did not significantly vary. As a result, the authors suggested that there may be methodological limitations in the use of RPE. These results highlight the need to be cautious when using subjective methods to study female basketball players, especially given the physiological differences from male players. It is necessary to conduct further studies on how hormonal levels may modify the perceived effort. Researchers and coaches must consider the physiological characteristics specific to female athletes when studying load through psychophysiological methods.

In addition to the limitations of RPE already noted, Foster’s method faces yet another limitation [66]. While this method is useful for quantifying internal load in practice, it becomes less reliable when applied to competitive events where the exposure time is shorter relative to training events. This can make it difficult to compare or mix load values between practice and games. For example, a 100-min practice session where a player reports an RPE of 5 (500 arbitrary units) will be higher than 40 min at an RPE of 10 (400 arbitrary units). To our knowledge, no solutions have been proposed to solve this aspect specific to basketball. To address this issue, one possibility could be to weigh the competition’s load values by a factor based on the characteristics of the population of interest. Further research is needed to fully understand and address this issue in basketball load monitoring.

### 4.2. Device-Based Methods for Internal Load Monitoring

As previously noted, limitations exist with respect to equipping basketball players with sensors during official matches, which could impede the study of their physiological responses. This could explain why we find more publications in young and amateur female players, where there may be less control over the use of these devices. Despite this, studies have been conducted evaluating the cardiac responses in professional and elite female players, indicating that there may be a relationship between a higher competitive level and increased internal response. This could be an indicator that physical demand is also higher. This relationship has already been described in the literature in many team sports, including women’s basketball [4,9].

HR monitoring is a widely adopted technique in team sports for evaluating the intensity, volume, and density of work [4,67]. However, its applicability to basketball may have the same limitations as other team sports [68]. The limitations of HR monitoring include the lag between neuromuscular activity and cardiovascular response and the requirement for extended exertion to produce significant cardiovascular activity. So, one optimal use of HR sensors is assessing the density of effort (ratio between activity and rest) in either a particular task or an entire training session [68]. Moreover, cardiac reaction to stress is an individualized parameter, and comparing individuals to averaged data could result in the misinterpretation of individual physiological responses. To avoid such errors, an individualized approach to monitoring should be adopted, considering the individual’s complete participation in different training tasks or competitions, avoiding mainly team sums and averages, including metrics relative to the individual maximum HR, such as %HR_Max_, %HR_Avg_, or the time spent in different zones. Notably, the included studies in this systematic review failed to explain how the HR_Max_ of the players was estimated.

These limitations of HR data in team sports could have a relevant impact when generating new indicators based on cardiac activity records. Examples of these new indicators are the TRIMP_B_ and the SHRZ [68]. The latter method could be a suitable alternative in the context of women’s basketball, as it weights the time spent by players in higher heart rate zones with a higher constant. Therefore, one minute at 150 bpm is not the same as one minute at 190 bpm. However, the question of how many HR zones is appropriate must be resolved. While the original Edwards’ SHRZ method proposed five thresholds (zone 1: 50–60%, zone 2: 60–70%, zone 3: 70–80%, zone 4: 80–90%, zone 5: >90%), in this systematic review, we have identified publications that use a different number of thresholds and, therefore, different percentages of HR (Table 3). One of the proposals was made by Reina et al. in 2019 [49] through six different zones. The interest of this division lies in dividing Edwards’ original zone 5 into 2: (1) zone 5: 90–95%, (2) zone 6: >95%. This would allow for discrimination if a player reaches values close to their maximum, since observing this requires sustained effort over time at a very high intensity. This relationship between external load and cardiac response has been described in basketball and other sports [4,9]. This hypothesis gains further support by examining the differences in the time spent by players in zone 6 (>95% HR_Max_) between 3v3 and 5v5 basketball formats. In the 3v3 format, which has a shorter game duration, fewer participants, more space, and fewer interruptions due to different foul and serve regulations [69], players spent approximately 85% of their time in zone 6. On the other hand, in the 5v5 format, which has a longer game duration, more players, less space, and more interruptions, the time spent in zone 6 did not even reach 10% [62]. These findings suggest that the demands of the game format influence the intensity and distribution of the players’ efforts during the game.

Finally, some authors indicate the importance of evaluating time-related cardiac responses to gain a more accurate understanding of the heart’s response to the time dose duration [49]. Such an approach may provide more precise insights into the physiological demands related to external load. The utilization of the useful time metric, as a quantification of effort, is most frequently applied in competitive settings, as discussed above about Foster’s method. In addition, in the case of official competitions, this systematic review found that players spent most of the time above 85% of their HR_Max_ [45,52,62]. However, extending the use of useful time to training sessions, and even isolated tasks, could offer significant insights. Such an approach could help to identify the intensity and distribution of effort during training. Furthermore, these data may inform the development of more effective training programs, helping coaches to optimize and improve the physical performance of female basketball players by targeting specific respiratory, metabolic, and/or neuromuscular aspects. Future research and consensus may resolve these limitations in the use of heart rate, providing specific knowledge related to the characteristics of women basketball players.

### 4.3. Other Device-Based Methods Used for Internal Load Monitoring

Our systematic review identified two additional methods for monitoring internal load in basketball players during training and competition: BLC and VO_2_ analysis. Although the number of publications was limited, these methods offer valuable insights into the physiological responses of athletes. However, they are also invasive and require specific expertise and equipment for their accurate application, including blood handling, instrument calibration, and exercise breaks. Nonetheless, these methods could provide valuable information for the precision training and performance optimization of basketball players.

The BLC confirms the energy system used, specifically the process of glycolysis. This byproduct of cellular activity has been used to understand muscle demand and fatigue [52]. The values obtained from competition show a range between 3.2–6.0 mmol·L^−1^, including 5v5 and 3v3 competitions. These values were similar to those found in male basketball players [11]. The observed range of lactate concentration during competition suggests that glycolysis is a significant contributor to energy production. This could be explained by the specific demands of basketball, such as repeated high-intensity actions and intermittent efforts. Although one included publication studied the effects of small-sided games in training [44], it did not report absolute lactate values, and therefore no reference values were obtained for these events. It could be speculated that the BLC values in training could be lower due to a lower density compared to competition. Therefore, this method might be better suited for specific tasks than for whole sessions.

BLC monitoring in basketball may provide valuable information about energy metabolism and muscle demand. The potential invasiveness of this method for monitoring internal load may discourage its use in some contexts. However, it may be possible to overcome these barriers as technology progresses. Compact, secure, and real-time monitoring sensors have been reported in the literature [70], which could offer a promising alternative for internal load monitoring. These advancements could facilitate the use of these metrics in decision-making processes in various settings. For this reason, more research is needed on the effects of training and competition on these types of metabolic markers, which will help us to better understand subsequent adaptation.

Finally, our systematic review identified few studies reporting VO_2_ in female basketball players, with only one study reporting values during games [35]. The reported values of 33.4 ± 4.0 mL/kg/min (66.7 ± 7.5% VO_2Max_) suggest moderate to high aerobic demands during competition, which could be important for designing effective training programs for female basketball players. Further research in this area is needed to fully understand the metabolic demands of women’s basketball.

### 4.4. Limitations

The variability in metrics used for internal load assessment in female basketball makes it challenging to compare results within and between events, competitive levels, and age groups. This issue has been identified in previous systematic reviews [7,14]. A potential explanation for the diverse metrics and values reported in the literature could be the publication of retrospective studies that lack a clearly defined methodology for data collection. As a result, the values obtained may not reflect the actual training dynamics. Many of the studies included in this review did not provide essential information on the dose of training or competition, research methodology, or data and participant loss during the process. To enhance the comprehensiveness of future research, it is recommended to include external load as a contextual factor when analyzing physiological responses.

## 5. Conclusions

This systematic review provides an overview of the methods and metrics used to measure internal load in female basketball players during both practice and competition. We identified the most commonly used approaches and highlighted the advantages and limitations of each. The findings suggest that future research in this area should prioritize the standardization of metrics and the development of objective, non-invasive measures of internal load. Additionally, coaches and practitioners should be aware of the limitations of subjective methods, such as RPE, and consider using multiple metrics to gain a more comprehensive understanding of internal load in female basketball players. Although this review provides a valuable reference framework for researchers and practitioners in this field, the use of multiple metrics with little standardization led to significant heterogeneity among the studies. Future research could address this limitation by adopting more consistent measurement protocols and standardizing the use of metrics. Overall, this review highlights the importance of accurately measuring internal load in female basketball players and provides a roadmap for future research in this area. Further research is needed to develop more objective measures of internal load and determine the optimal balance between external and internal load for improving athletic performance and preventing injury in this population.

## Data Availability

Not applicable.

## References

[1] Huyghe T., Alcaraz P.E., Calleja-González J., Bird S.P. (2022). The Underpinning Factors of NBA Game-Play Performance: A Systematic Review (2001–2020). Phys. Sportsmed..

[2] Weiss K.J., Allen S.V., McGuigan M.R., Whatman C.S. (2017). The Relationship Between Training Load and Injury in Men’s Professional Basketball. Int. J. Sports Physiol. Perform..

[3] Helwig J., Diels J., Röll M., Mahler H., Gollhofer A., Roecker K., Willwacher S. (2023). Relationships between External, Wearable Sensor-Based, and Internal Parameters: A Systematic Review. Sensors.

[4] Espasa Labrador J., Peña J., Caparrós Pons T., Cook M., Fort Vanmeerhaeghe A. (2021). Relationship between Internal and External Load in Elite Female Youth Basketball Players. Apunts Sports Med..

[5] Bourdon P.C., Cardinale M., Murray A., Gastin P., Kellmann M., Varley M.C., Gabbett T.J., Coutts A.J., Burgess D.J., Gregson W. (2017). Monitoring Athlete Training Loads: Consensus Statement. Int. J. Sports Physiol. Perform..

[6] Vanrenterghem J., Nedergaard N.J., Robinson M.A., Drust B. (2017). Training Load Monitoring in Team Sports: A Novel Framework Separating Physiological and Biomechanical Load-Adaptation Pathways. Sports Med..

[7] Russell J.L., McLean B.D., Impellizzeri F.M., Strack D.S., Coutts A.J. (2021). Measuring Physical Demands in Basketball: An Explorative Systematic Review of Practices. Sports Med..

[8] Elliott-Sale K.J., Minahan C.L., de Jonge X.A.K.J., Ackerman K.E., Sipilä S., Constantini N.W., Lebrun C.M., Hackney A.C. (2021). Methodological Considerations for Studies in Sport and Exercise Science with Women as Participants: A Working Guide for Standards of Practice for Research on Women. Sports Med..

[9] McLaren S.J., Macpherson T.W., Coutts A.J., Hurst C., Spears I.R., Weston M. (2018). The Relationships Between Internal and External Measures of Training Load and Intensity in Team Sports: A Meta-Analysis. Sports Med..

[10] Reina M., García-Rubio J., Ibáñez S.J. (2020). Training and Competition Load in Female Basketball: A Systematic Review. Int. J. Environ. Res. Public Health.

[11] Stojanović E., Stojiljković N., Scanlan A., Dalbo V., Berkelmans D., Milanović Z. (2018). The Activity Demands and Physiological Responses Encountered During Basketball Match-Play: A Systematic Review. Sports Med..

[12] Page M.J., McKenzie J.E., Bossuyt P.M., Boutron I., Hoffmann T.C., Mulrow C.D., Shamseer L., Tetzlaff J.M., Akl E.A., Brennan S.E. (2021). The PRISMA 2020 Statement: An Updated Guideline for Reporting Systematic Reviews. BMJ.

[13] Linder S.K., Kamath G.R., Pratt G.F., Saraykar S.S., Volk R.J. (2015). Citation Searches Are More Sensitive than Keyword Searches to Identify Studies Using Specific Measurement Instruments. Physiol. Behav..

[14] Petway A.J., Freitas T.T., Calleja-González J., Medina Leal D., Alcaraz P.E. (2020). Training Load and Match-Play Demands in Basketball Based on Competition Level: A Systematic Review. PLoS ONE.

[15] von Elm E., Altman D.G., Egger M., Pocock S.J., Gøtzsche P.C., Vandenbroucke J.P. (2007). The Strengthening the Reporting of Observational Studies in Epidemiology (STROBE) Statement: Guidelines for Reporting Observational Studies. PLoS Med..

[16] Anderson L., Triplett-McBride T., Foster C., Doberstein S., Brice G. (2003). Impact of Training Patterns on Incidence of Illness and Injury During a Women’s Collegiate Basketball Season. J. Strength Cond. Res..

[17] Scanlan A.T., Dascombe B.J., Reaburn P., Dalbo V.J. (2012). The Physiological and Activity Demands Experienced by Australian Female Basketball Players during Competition. J. Sci. Med. Sport.

[18] Coyne J.O.C., Nimphius S., Newton R.U., Gregory Haff G. (2019). Does Mathematical Coupling Matter to the Acute to Chronic Workload Ratio? A Case Study from Elite Sport. Int. J. Sports Physiol. Perform..

[19] Narazaki K., Berg K., Stergiou N., Chen B. (2009). Physiological Demands of Competitive Basketball. Scand. J. Med. Sci. Sports.

[20] Atl H., Köklü Y., Alemdaroğlu U., Koçak F.Ü. (2013). A Comparison of Heart Rate Response and Frequencies of Technical Actions Between Half-Court and Full-Court 3-A-Side Games in High School Female Basketball Players. J. Strength Cond. Res..

[21] Legg J., Pyne D., Semple S., Ball N. (2017). Variability of Jump Kinetics Related to Training Load in Elite Female Basketball. Sports.

[22] Azpiroz M.F., Feu S., Jiménez C., Calleja-GonzÁlez J. (2013). Perceived Exertion Effort in Mini Basketball Players and Its Relationship with Training Volume. Rev. Psicol. Deporte.

[23] Klusemann M.J., Pyne D.B., Foster C., Drinkwater E.J. (2012). Optimising Technical Skills and Physical Loading in Small-Sided Basketball Games. J. Sports Sci..

[24] Abad C.C.C., Pereira L.A., Kobal R., Kitamura K., Cruz I.F., Loturco I., Nakamura F.Y. (2016). Heart Rate and Heart Rate Variability of Yo-Yo IR1 and Simulated Match in Young Female Basketball Athletes: A Comparative Study. Int. J. Perform. Anal. Sport.

[25] Lastella M., Roach G.D., Vincent G.E., Scanlan A.T., Halson S.L., Sargent C. (2020). The Impact of Training Load on Sleep During a 14-Day Training Camp in Elite, Adolescent, Female Basketball Players. Int. J. Sports Physiol. Perform..

[26] Lukonaitienė I., Kamandulis S., Paulauskas H., Domeika A., Pliauga V., Kreivytė R., Stanislovaitienė J., Conte D. (2020). Investigating the Workload, Readiness and Physical Performance Changes during Intensified 3-Week Preparation Periods in Female National Under18 and Under20 Basketball Teams. J. Sports Sci..

[27] Otaegi A., Los Arcos A. (2020). Quantification of the Perceived Training Load in Young Female Basketball Players. J. Strength Cond. Res..

[28] Suárez Iglesias D., Dehesa R., Scanlan A., Rodríguez-Marroyo J., Vaquera A. (2020). Defensive Strategy and Player Sex Impact Heart Rate Responses during Games-Based Drills in Professional Basketball. Int. J. Sport. Physiol. Perform..

[29] Senbel S., Sharma S., Raval M.S., Taber C., Nolan J., Artan N.S., Ezzeddine D., Kaya T. (2022). Impact of Sleep and Training on Game Performance and Injury in Division-1 Women’s Basketball Amidst the Pandemic. IEEE Access.

[30] Reina M., García-Rubio J., Antúnez A., Courel-Ibáñez J., Ibáñez S.J. (2019). Load Variability of Training Sessions and Competition in Female Basketball. Rev. Psicol. Deporte.

[31] Montgomery P.G., Maloney B.D. (2018). Three-by-Three Basketball: Inertial Movement and Physiological Demands During Elite Games. Int. J. Sports Physiol. Perform..

[32] Vencúrik T., Nykodým J., Bokůvka D., Rupčić T., Knjaz D., Dukarić V., Struhár I. (2021). Determinants of Dribbling and Passing Skills in Competitive Games of Women’s Basketball. Int. J. Environ. Res. Public Health.

[33] Gutiérrez-Vargas R., Pino-Ortega J., Ugalde-Ramírez A., Sánchez-Ureña B., Blanco-Romero L., Trejos-Montoya J., Gutiérrez-Vargas J.C., Rojas-Valverde D. (2022). Physical and Physiological Demands According to Gender, Playing Positions, and Match Outcomes in Youth Basketball Players. RICYDE Rev. Int. Cienc. Deporte.

[34] Lupo C., Ungureanu A.N., Frati R., Panichi M., Grillo S., Brustio P.R. (2020). Player Session Rating of Perceived Exertion: A More Valid Tool Than Coaches’ Ratings to Monitor Internal Training Load in Elite Youth Female Basketball. Int. J. Sports Physiol. Perform..

[35] Sanders G.J., Boos B., Rhodes J., Kollock R.O., Peacock C.A., Scheadler C.M. (2019). Factors Associated with Minimal Changes in Countermovement Jump Performance throughout a Competitive Division I Collegiate Basketball Season. J. Sports Sci..

[36] Coyne J.O.C., Coutts A.J., Newton R.U., Gregory Haff G. (2021). Relationships Between Different Internal and External Training Load Variables and Elite International Women’s Basketball Performance. Int. J. Sports Physiol. Perform..

[37] Willberg C., Wellm D., Behringer M., Zentgraf K. (2023). Analyzing Acute and Daily Load Parameters in Match Situations–a Comparison of Classic and 3 × 3 Basketball. Int. J. Sports Sci. Coach..

[38] Sanders G.J., Boos B., Rhodes J., Kollock R.O., Peacock C.A. (2021). Competition-Based Heart Rate, Training Load, and Time Played Above 85% Peak Heart Rate in NCAA Division I Women’s Basketball. J. Strength Cond. Res..

[39] Delextrat A., Trochym E., Calleja-González J. (2012). Effect of a Typical In-Season Week on Strength Jump and Sprint Performances in National-Level Female Basketball Players. J. Sports Med. Phys. Fit..

[40] Ortega V., Suero E.J.F. (2017). Relationship between Technical-Tactical Complexity in the Training Session and Internal Load in Female Basketball. Sport TK-Rev. Euroam. Cienc. Deporte.

[41] Cruz I.d.F., Pereira L.A., Kobal R., Kitamura K., Cedra C., Loturco I., Cal Abad C.C. (2018). Perceived Training Load and Jumping Responses Following Nine Weeks of a Competitive Period in Young Female Basketball Players. PeerJ.

[42] Paulauskas H., Kreivyte R., Scanlan A.T., Moreira A., Siupsinskas L., Conte D. (2019). Monitoring Workload in Elite Female Basketball Players During the In-Season Phase: Weekly Fluctuations and Effect of Playing Time. Int. J. Sports Physiol. Perform..

[43] Staunton C., Wundersitz D., Gordon B., Kingsley M. (2020). Discrepancies Exist between Exercise Prescription and Dose in Elite Women’s Basketball Pre-Season. Sports.

[44] Brini S., Abderrahman A.B., Clark C.C.T., Zouita S., Hackney A.C., Govindasamy K., Granacher U., Zouhal H. (2021). Sex-Specific Effects of Small-Sided Games in Basketball on Psychometric and Physiological Markers during Ramadan Intermittent Fasting: A Pilot Study. BMC Sports Sci. Med. Rehabil..

[45] Vencúrik T., Nykodým J., Vacenovský P. (2016). Heart Rate Analysis of Semi-Elite Female Basketball Players during Competitive Games. Stud. Sport.

[46] Vala R., Valová M., Pacut M. (2019). Heart Rate Response Differs between Elite and Non-Elite Czech Female Basketball Matches. J. Phys. Educ. Sport.

[47] Piñar M.I., García D., Mancha-Triguero D., Ibáñez S.J. (2022). Effect of Situational and Individual Factors on Training Load and Game Performance in Liga Femenina 2 Basketball Female Players. Appl. Sci..

[48] Bastida Castillo A., Gómez Carmona C.D., De la cruz sánchez E., Pino Ortega J. (2018). Accuracy, Intra- and Inter-Unit Reliability, and Comparison between GPS and UWB-Based Position-Tracking Systems Used for Time–Motion Analyses in Soccer. Eur. J. Sport Sci..

[49] Reina Román M., García-Rubio J., Feu S., Ibáñez S.J. (2019). Training and Competition Load Monitoring and Analysis of Women’s Amateur Basketball by Playing Position: Approach Study. Front. Psychol..

[50] Kraft J.A., Laurent M.C., Green J.M., Helm J., Roberts C., Holt S. (2020). Examination of Coach and Player Perceptions of Recovery and Exertion. J. Strength Cond. Res..

[51] Sansone P., Tschan H., Foster C., Tessitore A. (2020). Monitoring Training Load and Perceived Recovery in Female Basketball: Implications for Training Design. J. Strength Cond. Res..

[52] Matthew D., Delextrat A. (2009). Heart Rate, Blood Lactate Concentration, and Time–Motion Analysis of Female Basketball Players during Competition. J. Sports Sci..

[53] Reina M., Mancha D., Feu S., Ibáñez S.J. (2017). Is Training Carried out the Same as Competition? Analysis of Load in Women’s Basketball. Rev. Psicol. Deporte.

[54] Vallés Ortega C., Fernández-Ozcorta E.J., Fierro Suero S. (2017). Fatigue-Recovery Pattern in a Competition Competitive High Density Junior Women’s Basketbal, Patrón Fatiga-Recuperación En Una Competición de Alta Densidad Competitiva En Baloncesto Femenino Juniors. Cuad. Psicol. Deporte.

[55] Batalla Gavaldà A., Bofill Ródenas A.M., Montoliu Colás R., Corbi Soler F. (2018). Relationship between Heart Rate and the Scoreboard during a Relegation Playoff. Apunts Educ. Física Deport..

[56] Batalla-Gavalda A., Beltran-Garrido J.V., Garrosa-Martín G., Cecilia-Gallego P., Montoliu-Colás R., Corbi F. (2022). Long-Term Analyses of the Rate of Perceived Exertion as an Indicator of Intensity in Women’s Basketball during a Relegation Play-Off. Biology.

[57] Messias L.H.D., Camargo B.F., Ferrari H.G., Cardoso J.P.P., Manchado-Gobatto F.B. (2017). Effect of Mathematical Modelling on Determining Lactate Minimum Test Parameters before and after Seven Weeks of Monitored Training. Sci. Sports.

[58] Nunes J.A., Moreira A., Crewther B.T., Nosaka K., Viveiros L., Aoki M.S. (2014). Monitoring Training Load, Recovery-Stress State, Immune-Endocrine Responses, and Physical Performance in Elite Female Basketball Players During a Periodized Training Program. J. Strength Cond. Res..

[59] Sanchez-Sanchez J., Carretero M., Valiente J., Gonzalo-Skok O., Sampaio J., Casamichana D. (2018). Heart Rate Response and Technical Demands of Different Small-Sided Game Formats in Young Female Basketballers. [Respuesta de La Frecuencia Cardíaca y Demanda Técnica En Diferentes Formatos de Juegos Reducidos Realizados Por Jugadoras Jóvenes de Baloncesto]. RICYDE Rev. Int. Cienc. Deporte.

[60] Reina M., Mancha-Triguero D., García-Santos D., García-Rubio J., Ibáñez S.J. (2019). Comparación de Tres Métodos de Cuantificación de La Carga de Entrenamiento En Baloncesto./Comparison of Three Methods of Quantifying the Training Load in Basketball. RICYDE Rev. Int. Cienc. Deporte.

[61] Andrianova R.I., Fedoseev D.V., Chicherin V.P., Lubyshev E.A., Krasilnikov A.A. (2021). Adaptation of the Training Process of Highly Qualified Women’s Basketball Teams Based on Indicators of Competitive Intensity and Calorie Consumption during Official Games. J. Phys. Educ. Sport.

[62] Reina M., García Rubio J., Antúnez A., José Ibáñez S. (2020). Comparison of Internal and External Load in Official 3 vs. 3 and 5 vs. 5 Female Basketball Competitions. Retos Nuevas Perspect. Educ. Física Deporte Recreación.

[63] Borg G. (1970). Perceived Exertion as an Indicator of Somatic Stress. Scand. J. Rehabil. Med..

[64] Halperin I., Emanuel A. (2020). Rating of Perceived Effort: Methodological Concerns and Future Directions. Sports Med..

[65] Paludo A.C., Paravlic A., Dvořáková K., Gimunová M. (2022). The Effect of Menstrual Cycle on Perceptual Responses in Athletes: A Systematic Review With Meta-Analysis. Front. Psychol..

[66] Foster C., Florhaug J.A., Franklin J., Gottschall L., Hrovatin L.A., Parker S., Doleshal P., Dodge C. (2001). A New Approach to Monitoring Exercise Training. J. Strength Cond. Res..

[67] Scanlan A.T., Wen N., Tucker P.S., Dalbo V.J. (2014). The Relationships between Internal and External Training Load Models during Basketball Training. J. Strength Cond. Res..

[68] Schneider C., Hanakam F., Wiewelhove T., Döweling A., Kellmann M., Meyer T., Pfeiffer M., Ferrauti A. (2018). Heart Rate Monitoring in Team Sports-A Conceptual Framework for Contextualizing Heart Rate Measures for Training and Recovery Prescription. Front. Physiol..

[69] Portes R., Jiménez S.L., Navarro R.M., Scanlan A.T., Gómez M.-Á. (2020). Comparing the External Loads Encountered during Competition between Elite, Junior Male and Female Basketball Players. Int. J. Environ. Res. Public Health.

[70] Tehrani F., Teymourian H., Wuerstle B., Kavner J., Patel R., Furmidge A., Aghavali R., Hosseini-Toudeshki H., Brown C., Zhang F. (2022). An Integrated Wearable Microneedle Array for the Continuous Monitoring of Multiple Biomarkers in Interstitial Fluid. Nat. Biomed. Eng..

